# Supplementary material for: Impact of the social context on the prognosis of Chagas disease patients: Multilevel analysis of a Brazilian cohort
Source: PLoS Negl Trop Dis. 2020 Jun 29;14(6):e0008399. doi: 10.1371/journal.pntd.0008399 (PMC7351237; doi:10.1371/journal.pntd.0008399)
Supplement: Attachment — Submitted filename: mpact of the social context on the prognosis of Chagas disease patients REVIEW.docx [file pntd.0008399.s003.docx]

That paper represents a good job performed by a group with consolidated high-quality research activity. Results could be considered very valuable as the approach used is quite novel and with a potential high impact among policy makers.

However, I believe that some aspects could be improved.

1.- First of all, the resolution of figures should be improved (pay also attention that the language used should be english!)

2.- 134 patients died, and authors have attributed as related to cardiovascular diseases. Is there any other information regarding the cause of death? Such as cancer, accident injuries, tuberculosis….. not precisely related to cardiovascular events or Chagas disease?

3.- **Table 2 (overall).** Probably this is the most relevant table/information among the paper, but to my opinion it is not well presented.

When variables are dichotomic, it is not needed to express both of them. I mean if want to calculate de proportion of any event among for example male, the number in female will be complementary.

Another point is to understand what do the authors wanted to calculate and how do the put it on the table. For example, in the case of the variable named “use of BNZ”

I understand that authors wanted to compare the proportion of patients whom used BNZ between those patients which developed cardiovascular events and those who did not.

As it is expressed, the proportions are calculated over the lines and not the columns

This is how it is showed now

| **1** |  | Cardiovascular event | | p |
| --- | --- | --- | --- | --- |
|  |  | Absent | Present |  |
| Use of BNZ | yes | 384 (93.8%) | 26 (6.3) | <p.001 |
|  | no | 953 (86.2) | 152 (13.8) |  |

Obviously the “p” is quite significant, but not sure which are the comparisons made (93.8 vs 6.3 or 93.8 vs 86.2) I hope it was the second one…

It is more correct (and easier to interpret) to express results like that:

| **2** |  | Cardiovascular event | | p |
| --- | --- | --- | --- | --- |
|  |  | Absent | Present |  |
| Use of BNZ | yes | 384/1337 (28.7%) | 26/179 (14.5%) | <p.001 |
|  |  |  |  |  |

In “senso estricto” proportions remain equal, but when try to read the results it is better to say that 28% of patients without cardiovascular event received bnz (number 2) instead of 93.8% of patients treated with BNZ did not developed cardiovascular event (number 1) It could resemble more sensationalist (which could led to the misunderstanding of the readers).

I hope I've made myself understood…..

4.- The age chosen to compare the presence of cardiovascular events was 60. That age is totally arbitrary without any justification. It would be more appropriate to compare the mean (or median) age and to use a T student.

5.- How do the authors explain the difference found regarding the marital status. Why to have a stable relationship seems to be protective against cardiovascular events????

6.- Line	364: It is said that “…individuals living in municipalities with a larger rural population showed protection against cardiovascular events, diverging from what would initially be expected..” Do not know which is the author’s background, but instinctively it is logical to understand that in larger rural municipalities better health care services were provided. Why do authors think contrary to that?

7.- Line 449: It is stated that “Not using BZN also increased the probability of experiencing cardiovascular events” If I am not wrong, when multilevel logistic regression model was performed, the use of BNS did not show any statistical difference (p 0.057). Isn’t it?
