# Supplementary material for: Impact of the social context on the prognosis of Chagas disease patients: Multilevel analysis of a Brazilian cohort
Source: PLoS Negl Trop Dis. 2020 Jun 29;14(6):e0008399. doi: 10.1371/journal.pntd.0008399 (PMC7351237; doi:10.1371/journal.pntd.0008399)
Supplement: Attachment — Submitted filename: letter_to_the_reviewer_.docx [file pntd.0008399.s004.docx]

**February, 26^st^, 2020.**

Dear Editor of Plos Neglected Disease,

Initially, on behalf of all the authors of this manuscript (PNTD-D-19-01738), I would like to thank you both reviewers for all contributions you have made for our manuscript. We do believe that the criticisms and suggestions were fundamental for improvement of our study.

We have described the criticisms and suggestions reported by each reviewer followed by the author´s answers and comments about them. All changes that have been made in the manuscript by the authors have been highlighted in the text of the manuscript in order to facilitate their identification.

| **(Reviewer 1)** | **Author´s answers** |
| --- | --- |
| General comments  That paper represents a good job performed by a group with consolidated high-quality research activity. Results could be considered very valuable as the approach used is quite novel and with a potential high impact among policy makers. However, I believe that some aspects could be improved. | Thank you for the kind comments. |
| 1- First of all, the resolution of figures should be improved (pay also attention that the language used should be english!) | Thanks for the comment. The figure initially attached was wrong. The recommendation has been accepted and a new figure attached, now with better resolution and in English. |
| 2.- 134 patients died, and authors have attributed as related to cardiovascular diseases. Is there any other information regarding the cause of death? Such as cancer, accident injuries, tuberculosis….. not precisely related to cardiovascular events or Chagas disease? | Thanks for the comment. We acknowledge that the use of all cause mortality is a limitation of the study. In general, deaths associated with Chagas disease are due to cardiovascular causes, mainly sudden death or progressive heart failure. In the present study, only 4 non-cardiovascular deaths occurred (one accidental death, two due to cancer and one non-specified death). However, as we were unable to assess the cause of death of each patient, all cause mortality was defined as the outcome.  This information has been added to the study limitation on page 22, lines 464-469 |
| 3- Table 2 (overall). Probably this is the most relevant table/information among the paper, but to my opinion it is not well presented.  When variables are dichotomic, it is not needed to express both of them. I mean if want to calculate de proportion of any event among for example male, the number in female will be complementary.  Another point is to understand what do the authors wanted to calculate and how do the put it on the table. For example, in the case of the variable named “use of BNZ”  I understand that authors wanted to compare the proportion of patients whom used BNZ between those patients which developed cardiovascular events and those who did not.  As it is expressed, the proportions are calculated over the lines and not the columns  This is how it is showed now      Obviously the “p” is quite significant, but not sure which are the comparisons made (93.8 vs 6.3 or 93.8 vs 86.2) I hope it was the second one…  It is more correct (and easier to interpret) to express results like that:      In “senso estricto” proportions remain equal, but when try to read the results it is better to say that 28% of patients without cardiovascular event received bnz (number 2) instead of 93.8% of patients treated with BNZ did not developed cardiovascular event (number 1) It could resemble more sensationalist (which could led to the misunderstanding of the readers).  I hope I've made myself understood….. | Thank you for the scientific orientation.  Suggestion accepted. Table 2 has been revised, following the recommendations (Results section, page 13). |
| 4.- The age chosen to compare the presence of cardiovascular events was 60. That age is totally arbitrary without any justification. It would be more appropriate to compare the mean (or median) age and to use a T student. | Thank you for the comment. We chose to work with the variable “age” in a dicotomic way because according to the WHO the age of 60 distinguishes adults from the elderly in developing countries. Thus, we chose to consider this variable as categorical in order to allow such a comparison. The WHO reference has been added (methods section, line 216-218). In addition, information has been added regarding the average age among those who had or did not have cardiovascular events (results section, line 295-297). |
| 5.- How do the authors explain the difference found regarding the marital status. Why to have a stable relationship seems to be protective against cardiovascular events???? | Thank you for the comment. We we base this discussion on the literature. Some studies have found that having a stable relationship is a protective factor for some diseases. Our comments in this regard are in the discussion session (lines 440-446), supported by references 43 and 44. |
| 6.- Line 364: It is said that “…individuals living in municipalities with a larger rural population showed protection against cardiovascular events, diverging from what would initially be expected..” Do not know which is the author’s background, but instinctively it is logical to understand that in larger rural municipalities better health care services were provided. Why do authors think contrary to that? | Thank you for the comment. We hypothesized that individuals residing in municipalities with a larger rural area would have greater difficulty in accessing health services due to long distances and difficulties in accessing places of care, in addition to commonly having less complex health services. However, considering the reflection raised, we have revised the wording and removed these comments from the manuscript, as well as the reference that accompanied it. (5th paragraph, discussion session, page 18)  Excerpt removed:  *diverging from what would initially be expected, given that in Brazil larger and more urban municipalities tend to have better health and care indicators [33].*  Reference removed:  33- Arruda, Natália Martins, Maia, Alexandre Gori e Alves, Luciana Correia. Desigualdade no acesso à saúde entre as áreas urbanas e rurais do Brasil: uma decomposição de fatores entre 1998 a 2008. Cadernos de Saúde Pública [online]. 2018, v. 34, n. 6 |
| 7.- Line 449: It is stated that “Not using BZN also increased the probability of experiencing cardiovascular events” If I am not wrong, when multilevel logistic regression model was performed, the use of BNS did not show any statistical difference (p 0.057). Isn’t it? | Thank you for the scientific orientation. We chose to keep two variables with a borderline p value in the final model (BZN use and marital status). The maintenance of these variables improved the fit of the final model. In addition, such variables have been recognized in previous studies [32; 43, 44; 48] as clinically relevant for CD.  Our option was also based on the limitations and criticisms pointed out by the scientific literature regarding the arbitrariness of the adoption of significance levels of 5 % with excess of rigidity (Kline, 2004; Rosnow and Rosenthal, 1989Chawla, 2017.  We have revised the wording of the excerpts that refer to such variables in the manuscript. We highlight the fact that the p value found was borderline, bringing greater caution in the interpretation of such findings (discussion session, Lines 330-331; lines 441- 443; lines 453-454).  Kline, R.B. (2004). Beyond significance testing: Reforming data analysis methods in behavioral research (2.ª ed.). Washington, DC: American Psychological Association.  Rosnow, R.L. e Rosenthal, R. (1989). Statistical procedures and the justification of knowledge in psychological science. The American Psychologist, 44(10), 1276-­‐1284  Chawla, Dalmeet Singh. 'One-size-fits-all’ threshold for P values under fire. Nature News. 2017. Avaiable in: <https://www.nature.com/news/one-size-fits-all-threshold-for-p-values-under-fire-1.22625> |

In accordance with the PLOS data access policy, we removed a note "data not shown".

We hope the modifications promoted in this manuscript have made our study more appropriate scientifically. However, the authors will be available to make any further changes that are still needed.

Sincerely,

Ariela Mota Ferreira
