# Supplementary material for: Impact of the social context on the prognosis of Chagas disease patients: Multilevel analysis of a Brazilian cohort
Source: PLoS Negl Trop Dis. 2020 Jun 29;14(6):e0008399. doi: 10.1371/journal.pntd.0008399 (PMC7351237; doi:10.1371/journal.pntd.0008399)
Supplement: Attachment — Submitted filename: letter_to_the_reviewer_2 (2).docx [file pntd.0008399.s005.docx]

**May, 04^th^, 2020.**

Dear Reviewer 2 of Plos Neglected Disease,

Initially, on behalf of all the authors of this manuscript (PNTD-D-19-01738), I would like to say sorry for not having sent the answer beforehand, along with the answer sent to Reviewer 1. Unfortunately due to our lack of attention, we did not identify Reviewer 2 in the first evaluation round. We are deeply sorry for our mistake. Here follows our return to this assessment.

We would like to thank you, Reviewer 2, for all contributions you have made for our manuscript. We do believe that the criticisms and suggestions were fundamental for improvement of our study.

Below we have shown the criticisms and suggestions reported by you, **(Reviewer 2**) followed by our answers and comments about them (**author´s answers**). All changes that have been made in the manuscript have been highlighted in the text in order to facilitate their identification. We emphasize that the changes made to the manuscript resulting from the contributions of Reviewer 1, also remain highlighted in the manuscript.

| **(Reviewer 2)** | **Author´s answers** |
| --- | --- |
| Lines 75 and 76. Authors speaks about social problem and social impact, based on data of reference 1. Please, explain what social impact means, because it is not explained into this reference, or alternatively, provide a reference to address it. | Thanks for the comment. The wording has been revised. The term “social impact” has been removed and replaced by information that CD represents one of the four leading causes of deaths from infectious and parasitic diseases in the world, with a reference that supports this statement. Lines 75-76 |
| Line 100. Authors said that CD is classically associated with poverty in rural areas (ref 11). I don’t agree with this statement given that in periurban areas of endemic countries, CD transmission (and prevalence) is even higher that in rural areas, due to internal migrant movements. I suggest authors to reformulate the statement | Thanks for the comment. The recommendation was accepted and the statement was removed. Line 100. |
| Study design. Why were negatives excluded from the analysis? How do you ensure that also negatives patients did not suffer from the same cardiological affections? I suggest authors to provide information about this group, if they have. If not, I consider this lack of information a study major limitation. | Thanks for the comment. We chose to exclude individuals with negative/inconclusive serology since the objective of our study was to evaluate factors that may interfere with the occurrence of cardiac events among individuals with CD. The analysis of the individuals with negative/inconclusive serology has been added (lines 296-298) (in addition to the analysis of the differential loss - lines 291- 296- that had previously considered such individuals).  Among the 161 individuals with negative/inconclusive serology, 10 (6 %) had a cardiac event (8 died, 1 developed atrial fibrillation and 1 required a pacemaker implant). This information has been added to the text (Lines 296-298). |
| Line 179: Theoretical model and variables. I think that this section should be better organized and included in Study design section | Thanks for the comment. The suggestion was accepted. The topic “theoretical model and variables” was reallocated, now becoming a subtopic of the “Study Design” session. Line 177. |
| In this same section (Theoretical model and variables), and even if authors include this information after, I suggest to provide information why (based on which data) AF and QRS complex duration were selected among arrhythmia types and EKG values. | Thanks for the comment. The suggestion was accepted. The wording has been revised.  Information was added on atrial fibrillation and pacemaker implantation. A new reference has been added (lines 182-195).  Regarding the *QRS complex duration*, the values observed on the ECG and the reference adopted are on lines 230-231. |
| Line 199: If I understood well, health related behavior variables were asked only at the beginning. From my point of view, it is possible that these value change during the follow-up period. Please provide information of this timepoint evaluation, and if you don’t have it, provide a rational why it was not considered to be included. | Thanks for the comment. In fact the information regarding the independent variables was all extracted from the baseline. This option was based on our interest in evaluating the data longitudinally (risk factors measured at baseline and outcome representing a new cardiac event that occurred between baseline and follow up). If we had included the independent variables analysis collected in the follow-up, we would have had a cross-sectional design and some inconsistent situations, since the events that made up the outcome of interest would have happened temporarily before the potential risk factor (for example, how would those who died be evaluated?).  In addition, we believe that adding extra information about how each variable has changed over time would be out of the focus of the study and would require an even larger text. |
| Line 206: why authors selected range 25-75 to dichotomize variables? Based on what? Please, provide more information on this decision. | Thanks for the comment. To make this decision, we performed some tests with the contextual variables, adopting different cutoff points to perform the categorization (mean, median, 25^th^ percentile and 75^th^ percentile). We observed that in general, the municipalities are relatively homogeneous and present predominantly unfavorable contexts. The dichotomization, that in our understanding best differentiated the municipalities, was to separate the 25 % of the municipalities in the best situation *vs* 75 % of the municipalities in the worst situation. Thus, we managed to highlight the small number of municipalities that presented a slightly better context than the others. This information is described on lines 210-217. |
| In case of the results, the analysis presented matched with the analysis plan. Results were mainly clearly presented. | Thanks for the comment. |
| Regarding self-reporting skin color, and given the high variety and variability of the variable. As a curiosity, why authors decide white/ non-white variables? | Thank you for the comment. Genetic studies conducted in Brazil have identified that there may be no significant association between skin color and genomic ancestry [1,2]. Thus, the skin color variable in Brazil reflects a socioeconomic aspect rather than a genetic aspect. Individuals who declare themselves oriental, black, mixed race or indigenous are similar in relation to socioeconomic aspects and at the same time differ from those who claim to be white. For this reason, categorization as white and non-white is recurrent in studies conducted in Brazil.   1. Parra FC, Amado RC, Lambertucci JR, Rocha J, Antunes CM, Pena SDJ. Color and genomic ancestry in Brazilians. Proc Natl Acad Sci USA. 2003; 100(1): 177-182. 2. Pimenta JR, Zuccherato LW, Debes AA, Maselli L, Soares RP, Moura-Neto RS, *et al*. Color and Genomic Ancestry in Brazilians: A Study with Forensic Microsatellites. Hum Hered. 2006; 62(4): 190-195. |
| Functional class: At /with limitations/ variable, did authors considered degree limitation? I think that information is scarce as dichotomized in this case. Please provide an explanation why the variable was established this way. | The functional class assesses tolerance to physical exertion, and is widely used to estimate the severity of heart failure.  In the context of Chagas disease, the functional class is a strong predictor of mortality, independent of other prognostic factors. However, the individual assessment of the degree of functional limitation is subjective, particularly in elderly, obese, pulmonary patients, and women. Thus for the present study in an endemic area, it was decided to dichotomize functional class I of the NYHA, which is characterized by the absence of dyspnea on exertion, comparing it with the other classes (classes II, III and IV), which classify dyspnea by the intensity of physical activity which is more influenced by the subjective character in the evaluation.  In fact functional classes III and IV are more advanced degrees of symptoms with a worse prognosis compared to the others. This information has been included in the study's limitations (Lines 485- 490). |
| Figure 1 should be translated into English | Thanks for the comment. The figure initially attached was wrong. The recommendation has been accepted and a new figure attached, now with better resolution and in English. |
| - Linha 324. 12% dos pacientes com DC tiveram eventos vasculares. Entre eles, quantos foram tratados? Por favor, forneça informações da análise dos pacientes por condição de tratamento  Line 324. 12% of the patients with CD had Cvascular events. Among them, how many were treated? Please, provide information of the analysis of patients by treatment condition | The specific treatment of CD in Brazil is conducted with the use of Benznidazole, and is described in Table 1. The treatment of cardiovascular events was not addressed because this is outside the focus of the study and the information is not available. |
| Lines 376-380. I think that this assumption is not based on the manuscript results presented. To provide information of this assumption was not the objective of the research, so there is no data to support this hypothesis. I suggest authors to reformulate or eliminate it. | We appreciate the comment and accept the suggestion to remove the assumption mentioned. (Line 383). |
| Same comment to 436-438 lines. | We appreciate the comment and accept the suggestion to remove the sentence mentioned. Line 438 |
| Line 453-454: there were people with altered NTproBNP without cardiovascular events? It seems clear that, as ventricular disfunction marker, proBNP is proven to be higher in older people. Please highlight if new information on this concern regarding this study results | NT-ProBNP was classified according to the age-specific cutoff points that define the diagnosis of heart failure. Thus, the effect of age on serum NT-ProBNP levels was considered (line 231).  Of the patients without cardiovascular events, 112 had abnormal NT-ProBNP values, indicating that other factors are associated with the occurrence of cardiovascular events.  However, we agree that ProBNP is a strong marker of ventricular dysfunction with a prognostic value. A previous study by the group showed that altered NT-ProBNP was the most important determinant of death during a 2 year period in this population of patients with Chagas disease [1]  1. Di Lorenzo Oliveira C, *et al.* Risk Score for Predicting 2-Year Mortality in Patients With Chagas Cardiomyopathy From Endemic Areas: SaMi-Trop Cohort Study. [J Am Heart Assoc.](https://www.ncbi.nlm.nih.gov/pubmed/32157953) 2020 Mar 17;9(6):e014176. |
| Antes de mais, gostaria de felicitar as pesquisas pela visão abrangente de que uma doença como Chagas é refletida nos resultados da pesquisa e no manuscrito.  No entanto, apesar da abordagem necessária e positiva, eu precisaria de um pouco mais de informação para chegar às conclusões dos autores.  First of all, I would like congratulate researches for the comprehensive view that a disease like Chagas, and that is reflected in the research results and the manuscript.  Nevertheless, and spite of the necessary and positive approach, I would have needed a little more information to arrive to authors’ conclusions. | Thanks for the comment. We hope we have been able to provide the requested information, and therefore improved the research approach. |

We add that in accordance with the PLOS data access policy, we removed a note "data not shown".

We hope the modifications promoted in this manuscript have made our study more appropriate scientifically. However, the authors will be available to make any further changes that are still needed.

Sincerely,

Ariela Mota Ferreira
